# Epidemiological features and prevalence patterns of prediabetes outcomes in an east coast Malaysian cohort: A retrospective cohort study

**DOI:** 10.51866/oa.994

**Published:** 2026-01-24

**Authors:** Nurul Azreen Yusof, Aniza Abdul Aziz, Nyi Nyi Naing, Nur Aiza Idris, Hassan Basri Mukhali, Muhd Zulfadli Hafiz Ismail, Rohaiza Abd Kadir, Mohamad Hasan Ahmad

**Affiliations:** 1 Faculty of Medicine, University Sultan Zainal Abidin, Jalan Sultan Mahmud, Kuala Terengganu, Malaysia.; 2 Biostatistic and Data Repository, National Institute of Health, Jalan Setia Murni U13/52, Seksyen U13, Setia Alam, Shah Alam, Selangor, Malaysia.; 3 Klinik Kesihatan Marang, Marang, Terengganu, Malaysia.; 4 Klinik Kesihatan Kerteh, Kerteh, Kemaman, Terengganu, Malaysia.

**Keywords:** Prediabetic state, Diabetes mellitus, Epidemiology, Health promotion, Lifestyle

## Abstract

**Introduction:**

The rising prevalence of prediabetes poses a significant public health challenge worldwide. This descriptive study aimed to offer insights into the epidemiological characteristics and progression patterns of prediabetes, which are crucial for informing effective interventions and addressing the growing diabetes mellitus (DM) epidemic.

**Methods:**

This retrospective cohort study involved 705 adults with prediabetes attending 28 health clinics in Terengganu, Malaysia, from January 2019 to June 2023. Data on sociodemographic characteristics, medical history and clinical measures during diagnosis and follow-up were collected from medical records and analysed using descriptive statistics. The progression trends to normoglycaemia, stable prediabetes and DM were determined based on glycated haemoglobin levels recorded over a 2-year follow-up period.

**Results:**

Of the 705 participants, 25.0% (n=176) reverted to normoglycaemia; 59.1% (n=4l7) remained stable with prediabetes; and 15.9% (n=112) progressed to DM within the 2-year follow-up. Dyslipidaemia, with higher triglyceride levels and lower high-density lipoprotein levels at diagnosis, was more prevalent among the participants who progressed to DM. Additionally, the participants with DM had higher median weight, body mass index and fasting blood sugar level at diagnosis than the other participants.

**Conclusion:**

This study underscores the critical need for targeted interventions to address the observed trends in the progression from prediabetes to DM in the Malaysian population. Comprehensive prediabetes programmes focusing on lifestyle modifications such as weight management, glycaemic control and lipid profile optimisation, along with regular monitoring, are essential in preventing progression to DM.

## Introduction

Diabetes mellitus (DM) ranks among the top global causes of death,^1^ accounting for over 80% of early NCD-related deaths along with cardiovascular disease, cancer and respiratory disease.^2^ The incidence of DM is reaching epidemic levels in low- and middle-income countries.^3,4^ It is contributed by the rising prevalence of prediabetes, a state of intermediate hyperglycaemia. In prediabetes, the glycaemic variables are elevated but not yet diabetic, and this leads to an increased risk of developing DM.^5^ Patients with impaired fasting glucose (IFG) and/or impaired glucose tolerance (IGT) based on oral glucose tolerance test (OGTT) results and/or glycated haemoglobin (HbAlc) levels of 5.7%–6.1% are classified as prediabetic.^6^ Prediabetes progresses to DM in about 25% of patients within 3–5 years, and as many as 70% of individuals with prediabetes eventually develop DM.^5,7^

Numerous studies reported the cardiovascular risk is increased in patients with prediabetes by 10%–40% compared with populations with normal glucose regulation.^8,9^ In 2019, the prevalence of prediabetes increased to 15.5%, affecting 3.4 million individuals worldwide, from 4.4% in 2010.^10^ Globally, the number of adults aged 20–79 years with IGT is projected to increase to 453.8 million or 8.0% of the adult population by 2030 and to 548.4 million or 8.6% of the adult population by 2045.^10^ Hence, prediabetes is becoming a global public health concern. According to the Malaysian National Health and Morbidity Survey (NHMS) 2023, the prevalence of DM among adults aged 18 years or older in Malaysia was increased to 15.6%,^11^ in contrast with the NHMS 2015 showing a lower prevalence of 13.4%.^12^ In Terengganu, the prevalence of DM exceeded the national figure, with the state rising from 18.6% in 2015 to 20.5% in 2019.^12^ A study conducted in Penang revealed that 10.1% of adults were diagnosed with prediabetes, while 19.6% were diabetic.^13^ Another large cohort study conducted in Malaysia in 2023 reported a prevalence of prediabetes and DM of 10.8% and 11.9%, respectively.^14^

Around 5%–10% of people with prediabetes become diabetic annually, though rates vary by population and definitions.^5^ In various major studies conducted globally, progression estimates have shown similarities. The annualised incidence was 11% in the Diabetes Prevention Program (DPP) Outcomes Study,^15^ 6% in participants with IFG in the United States (US) Multi-Ethnic Study of Atherosclerosis^16^ and 9% in participants with IFG and 7% in those with HbA1c levels of 5.7%-6.4% in a Japanese population-based study.^17^ A systematic review and meta-analysis reported that 3.7% of patients who had prediabetes had progressed to DM at 1-year follow-up. By 5 years, 17.1% had progressed to DM.^18^ Asians had the highest risk of progressing to DM and were nearly two times more likely to develop DM at 5 years than White people.^18^ A population-based longitudinal study in China in 2019 found that 41.3% of individuals remained prediabetic, while 11.0% of those with prediabetes progressed to DM at 12 years of follow-up.^19^ Another 5-year cohort study of 23,293 individuals with prediabetes in Spain showed that 40.9% had persistent prediabetes, while 23.0% progressed to DM.^20^

Older age, overweight, obesity and triglyceride level are independent predictors of an increased rate of progression to DM within 5 years.^14,18,20^ A retrospective cohort study in Sweden found that individuals with obesity and higher systolic blood pressure (SBP) at baseline had a higher probability of progressing to DM.^19^ A recent local study reported that participants who were older than 50 years, were of male sex, were of Malay race, were physically inactive, had hypertension or had a family history of DM had higher odds of having prediabetes and DM.^14^ Lower SBP and weight loss promoted the reversion from prediabetes to normoglycaemia, while obesity accelerated its progression to DM.^19^ The risk of progression to DM appeared to be lower in participants with a baseline glucose level of 5.6–6.1 mmol/L but increased substantially among those with a baseline glucose level of 6.2 mmol/L or higher.^18^ An HbA1c level of 6% or higher at the diagnosis of prediabetes was the strongest individual predictor of progression to DM at the 5-year follow-up.^20^ A large retrospective cohort study in the US found that a greater risk reduction in DM progression was achieved when patients with a baseline glucose level of 6.1 –6.6 mmol/L adhered to an intervention with resultant reduction in glucose levels. Reviews confirm lifestyle changes can delay DM progression. Therefore, thorough and early evaluation of any such interventions would be useful.^21-23^ According to local guidelines, patients with prediabetes require lifestyle changes and monitoring every 3-6 months.^6^

In Malaysia, the prevalence of DM has significantly increased, highlighting the urgency for national preventive strategies. Therefore, reversing prediabetes plays a significant role in reducing the incidence of DM and its related complications, and lower healthcare costs. Despite the extensive body of research conducted in high-income countries on the progression trends of prediabetes, there is a significant gap in understanding how these trends manifest in Malaysia, particularly given Malaysia’s unique context. Currently, no major studies have examined the progression of prediabetes within the Malaysian population. This gap limits the ability to develop targeted interventions tailored to Malaysia. Therefore, this study aimed to bridge the knowledge gap by estimating the prevalence of reversion to normoglycaemia, persistence of prediabetes and progression to DM among adults in Terengganu over a 2-year follow-up period. Additionally, we aimed to assess progression patterns in relation to demographic and clinical traits. The findings are crucial for informing local prevention strategies and laying the groundwork for targeted interventions to reduce the growing burden of DM in Malaysia.

## Methods

### Study design, sampling and participants

This retrospective cohort study involved 705 adults with prediabetes attending 28 health clinics (15 urban, 13 rural) in Terengganu from January 2019 to June 2023. As part of a broader investigation into DM progression, multiple sample size calculations were performed to meet different study objectives. For estimating prevalence, a single-proportion formula yielded a sample of 218 (13% progression rate, 5% precision, 5% significance). The largest required sample, 705, was based on smoking status (P0 = 19.4%, P1 = 26.4%) using a two-proportion formula, 80% power and 5% significance, including 20% dropout.

Inclusion criteria were adults (>18 years) diagnosed with prediabetes (2019–2021). Exclusion criteria included prior DM diagnosis, missing HbA1c data and loss to follow-up. Due to limited eligible patients and high dropout risk, no probability sampling was used. Participants were proportionally allocated across clinics based on prediabetes case volume.

### Measurement tools

Data were extracted from clinic records (2019–2021) and followed until June 2023. Trained healthcare professionals used standardised protocols, with independent verification by two reviewers. Discrepancies were resolved by consensus. A structured proforma captured sociodemographic, medical and clinical variables, including BMI, blood pressure, OGTT, lipid level and HbA1c. Glycaemic outcomes within 2 years were categorised as reversion to normoglycaemia, stable prediabetes or progression to DM.

The glycaemic categories used in this study were defined according to the Malaysian Clinical Practice Guidelines (CPG) on the Management ofType 2 Diabetes Mellitus (2020).^24^ The HbA1c cut-off values in the Malaysian CPG differ from those recommended by international guidelines such as the World Health Organization (WHO) and the American Diabetes Association (ADA) which define diabetes mellitus using an HbA1c threshold of >6.5%. The Malaysian CPG adopts a lower cut-off of HbA1c >6.3% in accordance to local studies in multi-ethnic Malaysian populations, which showed that an HbA1c level of 6.3% offers a better balance of sensitivity and specificity for diagnosing diabetes. Therefore, the HbA1c classification applied in this study reflects a locally validated, context-specific approach rather than direct reliance on international guideline cut-offs.

### Variable definitions

1. Normoglycaemia: Patients with prediabetes who reverted to normoglycaemia based on repeated HbA1c results (≤5.6%)^24^ (within 2 years of follow-up).
2. Stable prediabetes: Patients with prediabetes who remained at the prediabetic state based on repeated HbA1c results ranging from 5.7% to 6.2%^24^ (within 2 years of follow-up).
3. Prediabetes progression: Patients who progressed from prediabetes to DM based on repeated HbA1c results of 6.3% or more^24^ (within 2 years of follow-up).
4. Family history of DM: Patients who had a first-degree family history of DM.
5. Smoking status: Patients who were recorded as a smoker in the prediabetes record.
6. Underlying hypertension: Patients with pre-existing hypertension with or without medication.
7. Underlying dyslipidaemia: Patients with pre-existing dyslipidaemia with or without medication.
8. Weight: Patients’ body weight (kg) recorded at the diagnosis of prediabetes.
9. BMI: BMI (kg/m^2^) recorded at the diagnosis of prediabetes.
10. Fasting blood sugar (FBS) level: Level of venous blood glucose in the fasting state taken at diagnosis.
11. 2-h postprandial (2-HPP) glucose level: Level of venous blood glucose after 2 h of taking a standard 75-g glucose solution (at diagnosis).
12. Systolic blood pressure (SBP): SBP (mmHg) recorded at the diagnosis of prediabetes.
13. Diastolic blood pressure (DBP): DBP (mmHg) recorded at the diagnosis of prediabetes.
14. Cholesterol level: Levels of total cholesterol, triglycerides, low-density lipoprotein (LDL) cholesterol and high-density lipoprotein (HDL) cholesterol taken at diagnosis.
15. HbAlc level: Level of HbAlc (%) taken within 2 years of follow-up (but after at least 6 months of diagnosis to allow sufficient time for diet and lifestyle modifications).

### Statistical analysis

All statistical analyses were performed using Stata software version 17 (StataCorp LLC, College Station, TX, US). The baseline characteristics of adults with prediabetes were expressed as frequencies (percentages) for categorical data and medians (interquartile ranges [IQRs]) for skewed numerical data. The prevalence of reversion to normoglycaemia, stable prediabetes and progression to DM within 2 years among adults in Terengganu was estimated using descriptive statistics, specifically percentages and their 95% confidence intervals (CIs). The median weight, BMI, blood glucose level, blood pressure and cholesterol level were compared across three groups: normoglycaemia, prediabetes and DM. The significance of differences across these groups was determined using the Kruskal—Wallis test for continuous data, with the significance level set at 0.05. For categorical variables, Pearson’s chi-square test or Fisher’s exact test (as appropriate) was employed. Cases with missing values for a given variable were excluded from the relevant analysis, and this is indicated in the results tables (with denominators provided).

## Results

### Prevalence of normoglycaemia, stable prediabetes and DM

A total of 705 patients with prediabetes were included in this study. Among them, 25.0% (n=176, 95% CI=21.80–28.15) reverted to normoglycaemia; 59.1% (n=417, 95% CI=55.52–62.77) remained stable with prediabetes; and 15.9% (n=112, 95% CI=13.18–18.58) progressed to DM within 2 years of follow-up.

### General and medical background

The proportion of women was larger than that of men across all three groups (Table 1). The median (IQR) age was almost similar across the groups: 59 (22) years in the normoglycaemia group, 62 (16) years in the prediabetes group and 61.5 (15) years in the DM group. The majority of the participants were married and unemployed in all three groups. Non-smokers were predominant across all groups. The prevalence of dyslipidaemia was significantly higher in the DM group (88.4%, n=99) than in the prediabetes (84.9%, n=354) and normoglycaemia groups (72.2%, n=127). The prevalence of a family history of DM was slightly higher in the DM group (39.3%, n=44) than in the normoglycaemia (37.5%, n=66) and stable prediabetes groups (33.3%, n=139).

**Table 1:** Sociodemographic characteristics and medical background of the participants according to their glycaemic outcomes (N=705).

Variable	Frequency (%)			P-value^*^
	Normoglycaemia (n=176)	Stable prediabetes (n=417)	DM (n=112)	
**Sociodemographic characteristic**				
Race				0.875
Malay	174 (98.9)	405 (97.1)	110 (98.2)	
Chinese	2 (1.1)	10 (2.4)	2 (1.8)	
Indian	0 (0.0)	1 (0.2)	0 (0.0)	
Others	0 (0.0)	1 (0.2)	0 (0.0)	
Median (IQR) age, year	59 (22)	62 (16)	61.5 (15)	0.014
Sex				0.229
Male	46 (26.1)	137 (32.9)	37 (33.0)	
Female	130 (73.9)	280 (67.1)	75 (67.0)	
Marital status				0.505
Single	2 (1.1)	4 (1.0)	1 (0.9)	
Married	165 (93.8)	384 (92.1)	99 (88.4)	
Widowed	9 (5.1)	29 (7.0)	12 (10.7)	
Employment status				0.349
Unemployed	131 (74.4)	289 (69.3)	76 (67.9)	
Employed	45 (25.6)	128 (30.7)	36 (32.1)	
**Medical background**				
Smoking status				0.666
No	158 (89.8)	372 (89.2)	98 (87.5)	
Yes	18 (10.2)	45 (10.8)	14 (12.5)	
Underlying hypertension				0.067
No	34 (19.3)	61 (14.7)	9 (8.0)	
Yes	142 (80.7)	356 (85.3)	103 (92.0)	
Underlying dyslipidaemia				<0.001
No	48 (27.3)	59 (14.2)	11 (9.8)	
Yes	128 (72.7)	358 (85.8)	101 (90.2)	
Family history of DM				0.527
No	110 (62.5)	278 (66.7)	68 (60.7)	
Yes	66 (37.5)	139 (33.3)	44 (39.3)	

* P-values were calculated using Pearson’s chi-square test or Fisher’s exact test for categorical variables and the Kruskal-Wallis test for continuous variables. DM: diabetes mellitus

### Clinical characteristics

The DM group had significantly higher weight (median=69.6 kg; IQR= 17.88) and BMI (median=28.8 kg/m^2^; IQR=6.70). SBP and DBP showed no significant differences across the groups. The median FBS level (from the OGTT at the diagnosis of prediabetes) was higher in the DM group than in the other groups. However, no significant difference was observed in the 2-HPP glucose level across the groups. The median triglyceride level was significantly higher in the DM group (1.30; IQR=0.86), while the median HDL level was significantly lower in the DM group (1.35; IQR=0). There were no significant differences in the median total cholesterol and LDL levels between the groups. The median HbA1c level during follow-up was significantly higher in those who progressed to DM (6.45%; IQR=0.30) than in those who reverted to normoglycaemia (5.40%; IQR=0.40) and remained prediabetic (5.90%; IQR=0.30) (Table 2).

**Table 2:** Clinical characteristics of the participants according to their glycaemic outcomes within 2 years of follow-up (N=705).

Variable	Median (IQR)			P-value^*^
	Normoglycaemia (n=176)	Stable prediabetes (n=417)	DM (n=112)	
Weight (kg)	64.25 (17.75)	67 (16.05)	69.55 (17.88)	0.004
BMI (kg/m^2^)	26.55 (6.38)	27.8 (6.60)	28.8(6.70)	0.002
SBP (mmHg)	137 (21)	137 (18)	137(17)	0.633
DBP (mmHg)	80 (15)	80 (13)	82 (12)	0.777
FBS level (mmol/L) (n=598)	5.8 (1)	6.0 (1)	6.2 (1)	<0.001
2-h postprandial glucose level (mmol/L) (n=592)	8.80 (2)	8.90 (2)	8.60 (2)	0.723
Total cholesterol level (mmol/L)	5.60 (1.6)	5.31 (1.6)	5.68 (1.72)	0.204
Triglyceride level (mmol/L)	1.01 (0.69)	1.20 (0.70)	1.30 (0.86)	0.003
LDL level (mmol/L)	3.48 (1.60)	3.39 (1.50)	3.59 (1.68)	0.260
HDL level (mmol/L)	1.48 (1)	1.40 (0)	1.35 (0)	0.013
HbA1c level (%)	5.40 (0.40)	5.90 (0.30)	6.45 (0.30)	<0.001

IQR: interquartile range, BMI: body mass index, SBP: systolic blood pressure, DBP: diastolic blood pressure, FBS: fasting blood sugar, LDL: low-density lipoprotein, HDL: high-density lipoprotein, HbAlc: glycated haemoglobin.

* P-values were calculated using the Kruskal—Wallis test for continuous variables.

## Discussion

### Prevalence of reversion to normoglycaemia, stable prediabetes and progression to DM within 2 years of follow-up

This study revealed that out of the 705 patients with prediabetes, more than half (59.1%) remained in the prediabetic state; 25.0% reverted to normoglycaemia; and 15.9% progressed to DM within the 2-year follow-up. A significant proportion of patients with prediabetes remained in the IGT state, highlighting regular monitoring and early interventions to prevent progression to DM. Similar results were found in the previous studies conducted by Bennasar-Veny et al. in Spain,^20^ Wutthisathapornchai and Lertwattanarak in Thailand^25^ and Shang et al. in Sweden.^19^ Bennasar-Veny et al. reported that 40.9% of the population with prediabetes remained prediabetic over a 5-year period.^20^ A cohort study in Thailand found that 19.4% of participants with prediabetes developed DM within 3 years, with a mean time to progression to DM of 25.5 months.^25^ Similarly, a 12-year cohort study in Sweden revealed that 13% of patients with prediabetes progressed to DM, while 22% reverted to normoglycaemia, indicating the dynamic nature of glucose tolerance states.^19^ Moreover, Dejesus et al. showed that the 1- and 5-year DM incidence rates among individuals with prediabetes were 38.6 and 40.24 per 1000 person-years, respectively.^18^ These rates underscore the significant risk of progression from prediabetes to DM and the need for targeted interventions.

The observed regression rate of 25.0% in our study is consistent with the findings of Bennasar-Veny et al. and Shang et al.^19,20^ Approximately 36.1% of Spanish workers with prediabetes returned to normoglycaemia over a 5-year period. This study emphasised that physical activity, diet and a healthy BMI are critical factors influencing the persistence of prediabetes and progression to DM.^20^ Similarly, Shang et al. found that nearly 30% of older adults with prediabetes reverted to normoglycaemia during their study period. Specifically, their results showed a reversion rate of 22.2% over a 12-year follow-up, which is comparable to our findings.^19^ Another study reported that regression from prediabetes to normal glucose regulation was associated with a 56% reduction in DM incidence over 10 years.^15^ These studies highlight the significant role of lifestyle interventions in promoting regression to normoglycaemia and preventing DM progression.

### Sociodemographic characteristics

The demographic analysis showed a larger proportion of women in all three groups, consistent with other reports indicating sex-specific prevalence in glucose metabolism disorders.^13^ In our study, 67% of the participants who progressed to DM were women, and 33% were men. This aligns with the findings of Yeboah et al. in their multi-ethnic cohort study, showing that female participants with IFG were more likely to develop DM over 7.5 years of follow-up.^16^ These suggest that sex-specific biological and metabolic factors might influence the progression from prediabetes to DM. In the current study, the median age of the participants was similar across the groups, suggesting that age may not be a primary factor in the progression from prediabetes to DM within this cohort.

However, Bennasar-Veny et al. reported that age was a significant factor, with older individuals showing higher rates of progression to DM.^20^ Similarly, a large cohort study in Malaysia found that participants who were older than 50 years and male participants had increased odds of having prediabetes and DM.^14^ Other cohort studies in the US and Iran also identified older age as an independent predictor of an increased rate of progression to DM.^18,26^

Conversely, a large multi-ethnic cohort study conducted in the US from 2000 to 2022 showed that participants with IFG who developed DM over a 7-year period were slightly younger (mean age=62±10.0 years) than those who did not develop DM (mean age=65±9.6 years). However, further analysis indicated that age was not significant.^16^ This discrepancy highlights the need for age-stratified analyses in future research.

Most participants were married, unemployed and non-smokers, indicating common socioeconomic characteristics among the study population. In our study, most of the patients with prediabetes who progressed to DM were non-smokers. This finding is intriguing given the substantial body of research indicating strong associations between cigarette smoking, subsequent development of hyperglycaemia and insulin resistance, which consequently increase the risk of DM. For instance, the comprehensive review by Maddatu et al. highlighted various population-based studies in the US and Korea that linked cigarette smoking with a higher risk of developing DM. This review discussed mechanisms through which smoking and nicotine exposure affected insulin sensitivity and pancreatic β-cell function, thereby increasing the risk of DM.^27^ The discrepancy between our findings and the broader literature could be due to several factors. The lifestyle and metabolic factors in our study population could overshadow the effects of smoking on DM progression. Our study’s focus on patients with prediabetes might reveal different dynamics compared with studies focusing on the general population or individuals with different health profiles.

### Medical comorbidities

Dyslipidaemia and a family history of DM were more prevalent in the participants who progressed to DM, suggesting that these factors increase the risk of DM. Dyslipidaemia was significantly more prevalent in the DM group (88.4%) than in the stable prediabetes (84.9%) and normoglycaemia groups (72.2%). This finding aligns with existing literature that identifies dyslipidaemia, particularly hypertriglyceridaemia, as a major risk factor for DM.^13,15,20^ However, this contradicts the report by Shang et al., which did not identify a high total cholesterol level as a significant factor in DM progression.^19^ One possible reason for this discrepancy could be differences in the study populations and methodologies. Shang et al.’s study focused on older adults, who may have different lipid metabolism and cardiovascular risk profiles compared with a more diverse age group. Variations in dietary habits, genetic predispositions and other comorbidities might contribute to the differing impacts of dyslipidaemia on DM progression. Further research is needed to explore these factors and clarify the role of dyslipidaemia in DM development across different populations.

Our study found that a family history of DM was slightly more prevalent in the participants who progressed to DM (39.3%) than in those who reverted to normoglycaemia (37.5%) and remained prediabetic (33.3%). Previous studies have also consistently demonstrated that a family history of DM is a significant predictor of the progression from prediabetes to DM. In several DM risk models used in Australia, Europe and the US, a family history of DM was included as a component for predicting the risk of DM progression.^5^ This underscores the crucial role genetic predisposition plays in DM development, to consider family medical history in risk assessments and preventive strategies in prediabetes.^5,13^ It also highlights the importance of targeted screening and early intervention, suggesting that individuals with a family history of DM should be regarded as high-risk and monitored closely for signs of glucose dysregulation.

### Clinical characteristics

This study further examined the clinical characteristics of the participants to understand the factors contributing to the progression from prediabetes to DM. The analysis revealed significant differences in several clinical metrics between the three groups: those who progressed to DM, those who remained stable in the prediabetic state and those who reverted to normoglycaemia. The DM group had significantly higher median weight and BMI than the other groups. These findings highlight the observed trend of elevated body weight and BMI with the progression to DM, consistent with the results of other cohort studies.^18,20^ Janghorbani and Amini similarly reported that participants who progressed to DM had higher age-adjusted mean weight, BMI, waist circumference and hip circumference at baseline.^26^ The strong link between increased body weight and DM development can be attributed to several physiological mechanisms. Higher adiposity, particularly central obesity, is known to contribute to insulin resistance, a key factor in the pathogenesis of DM. Adipose tissue, especially when concentrated around the abdomen, releases free fatty acids and inflammatory cytokines that interfere with insulin signalling pathways, exacerbating hyperglycaemia and accelerating the transition from prediabetes to DM.^28^

Regarding blood pressure, our study did not observe notable differences in SBP and DBP among the individuals who remained prediabetic, reverted to normoglycaemia or progressed to DM over the 2-year follow-up period. This observation is consistent with findings reported in several other studies.^29,30^ A prospective cohort study on a Chinese population in 2021 found that changes in blood pressure were not significant among individuals who reverted from prediabetes to normoglycaemia compared with those who progressed to DM.^29^ The Whitehall II cohort study reported that while reversion from prediabetes to normoglycaemia was associated with improved cardiovascular outcomes, the differences in SBP and DBP were not significant in patients with prediabetes who reverted to normoglycaemia compared with those who progressed to DM.^30^ Suggesting blood pressure may not be a key factor in the metabolic transitions observed in prediabetes and DM progression. Other factors such as lifestyle and metabolic parameters might be more relevant in the progression or regression of prediabetes and DM.^30^ In contrast, other studies have indicated that a lower SBP is linked with a higher probability of reverting to normoglycaemia, implying that maintaining a lower SBP might facilitate the restoration of normal glucose regulation.^21^ Janghorbani and Amini observed that 24.6% of participants who progressed to DM had hypertension, compared with 17.1% of those who did not progress.^26^

The median FBS level at the diagnosis of prediabetes was higher in the DM group than in the other two groups, emphasising the importance of early blood sugar control. Similarly, a few other studies have highlighted the correlation between higher baseline FBS levels and the risk of progression to DM.^19,20,26^ Liu et al. reported that participants who progressed to DM had significantly higher mean FBS levels (6.27±0.39 mmol/L) than those who remained prediabetic (6.04±0.34 mmol/L) and those who reverted to normoglycaemia (5.96±0.32 mmol/L).^29^ Another cohort study in Iran showed that participants who progressed to DM had higher FBS and random plasma glucose levels at 30, 60 and 120 min at baseline, that could be explained by progressive β-cell failure, which is required for the deterioration of glucose homoeostasis and development of hyperglycaemia.^26^

One of the most striking findings from our study is the lipid profile outcomes. The participants who progressed to DM exhibited markedly higher median triglyceride levels and lower median HDL levels. There were no significant differences in the median total cholesterol and LDL levels across the three groups. These findings are consistent with previous reports highlighting the role of dyslipidaemia, particularly high triglyceride levels and low HDL levels, in the progression of DM.^13,20,26^ A prospective cohort study in a large Chinese population in 2021 found that participants who reverted from prediabetes to normoglycaemia had lower total cholesterol, triglyceride and LDL cholesterol levels than those who progressed to DM. Triglycerides and HDL cholesterol fall under the cluster of metabolic syndrome, which is one of the crucial factors associated with insulin resistance and DM progression.^31^ Reversion to normoglycaemia is associated with a reduced risk of cardiovascular events and ischaemic stroke.^29^ Therefore, effective management of cholesterol levels is crucial for improving glycaemic status and reducing the cardiovascular risk in individuals with prediabetes.^29^

Previous studies have highlighted the role of HbA1c levels in predicting the progression from prediabetes to DM.^18,19^ In our cohort study, the median HbA1c level during follow-up was significantly higher in the participants who progressed to DM (6.45%; IQR=0.30) than in those who reverted to normoglycaemia (5.40%; IQR=0.40) and remained prediabetic (5.90%; IQR=0.30). Higher HbA1c levels indicate poorer long-term glucose control and are a strong predictor of DM progression, reinforcing the findings by Dejesus et al., Shang et al. and Janghorbani and Amini that elevated HbA1c levels are a significant risk factor for developing DM.^18,19,26^ These clinical characteristics provide valuable insights into the risk factors associated with the progression from prediabetes to DM.

Implementing targeted interventions that incorporate comprehensive lifestyle modification programmes to effectively prevent the progression ofprediabetes to DM in the Malaysian population. These programmes should prioritise dietary improvements, increased physical activity and weight management.^20^ Structured initiatives similar to the DPP in the US, which has successfully reduced DM incidence by 58% over 3 years through lifestyle changes, could serve as a valuable model for implementation.^32^ Interventions focused on managing weight, enhancing fasting glucose control and optimising blood pressure and lipid profiles are crucial in mitigating the risk of DM progression among individuals with prediabetes.^18,19^ Integrating regular follow-up visits and community-based support groups could help sustain these lifestyle modifications. Implementing such comprehensive programmes in Malaysia could yield substantial public health benefits. Currently, the healthcare focus in Malaysia is predominantly on managing DM rather than addressing it at the prediabetic stage. Thus, it is critical to strengthen prediabetes programmes in health clinics to lower the future incidence of DM, reduce related morbidity and mortality and shift the focus from treatment to proactive early intervention.

This study has several limitations. First, the use of a non-probability sampling method limits the generalisability of the findings beyond the study population in Terengganu. Second, as a retrospective record-based study, the analysis was dependent on the accuracy and completeness of the medical records, and data on some relevant variables such as dietary habits, physical activity or socioeconomic status were not available. Third, the follow-up period was limited to 2 years, that may not capture longer-term patterns of reversion or progression. Finally, as the cohort was drawn from patients attending government health clinics, the findings may not be fully representative of individuals managed in private healthcare settings or those who do not seek regular follow-up. Despite these limitations, the study provides valuable insights into local epidemiological trends of prediabetes outcomes in Malaysia. It is one of the larger cohort studies of prediabetes conducted in Malaysia, involving 705 participants across 28 health clinics, which enhances the robustness of the findings. The study also benefited from systematic data extraction and verification by trained healthcare professionals, reducing the risk of measurement error. Importantly, the 2-year follow-up provided real-world evidence on progression and reversion trends, filling a critical gap in local epidemiological data on prediabetes.

## Conclusion

This study highlights the urgent need for targeted interventions to prevent the progression from prediabetes to DM in Malaysia. Lifestyle changes focusing on weight, glycaemic control, and lipid optimisation are essential and should be integrated into community programmes and national policies. A shift toward early intervention and comprehensive management of prediabetes is crucial to reducing DM rates and improving long-term outcomes. Future research should evaluate the real-world implementation and effectiveness of these strategies across diverse Malaysian populations.

## Data sharing statement

The raw data used in this study are not publicly available, as they contain sensitive patient information. However, the dataset was shared with the study statistician via encrypted, passwordprotected files to ensure data confidentiality and security. The data have not been deposited in any public repository. Access to the data may be granted upon reasonable request only to the corresponding author, subject to institutional and ethical approval.

**How does this paper make a difference in general practice?**

- Approximately 59.1% of patients remained prediabetic after 2 years, highlighting the need for structured follow-up in primary care—an area often overlooked in prior Malaysian studies.
- This study identifies key local risk factors for diabetes progression, including elevated BMI, high triglycerides, low HDL, and raised FBS and HbA1c levels, offering practical risk stratification for general practitioners.
- It addresses a major gap in Malaysian prediabetes cohort data, providing more accurate, context-specific guidance than international extrapolations.
- The findings support early lifestyle interventions and advocate a shift from reactive to preventive care, reinforcing current recommendations with new, locally relevant longitudinal evidence.
